# Mendelian randomization studies of depression: evidence, opportunities, and challenges

**DOI:** 10.1186/s12991-023-00479-6

**Published:** 2023-11-23

**Authors:** Wang-ran Ma, Lei-lei Zhang, Jing-ying Ma, Fang Yu, Ya-qing Hou, Xiang-rui Feng, Lin Yang

**Affiliations:** 1 Xian Hospital of Traditional Chinese Medicine, Xi’an, 710021 China; 2 Shanxi University of Traditional Chinese Medicine, Xianyang, 712046 China

**Keywords:** Causality, Instrumental variables, Mendelian randomization study, Depression, Risk factors

## Abstract

**Background:**

Major depressive disorder (MDD) poses a significant social and economic burden worldwide. Identifying exposures, risk factors, and biological mechanisms that are causally connected to MDD can help build a scientific basis for disease prevention and development of novel therapeutic approaches.

**Methods:**

In this systematic review, we assessed the evidence for causal relationships between putative causal risk factors and MDD from Mendelian randomization (MR) studies, following PRISMA. We assessed methodological quality based on key elements of the MR design: use of a full instrumental variable analysis and validation of the three key MR assumptions.

**Results:**

We included methodological details and results from 52 articles. A causal link between lifestyle, metabolic, inflammatory biomarkers, particular pathological states and MDD is supported by MR investigations, although results for each category varied substantially.

**Conclusions:**

While this review shows how MR can offer useful information for examining prospective treatment targets and better understanding the pathophysiology of MDD, some methodological flaws in the existing literature limit reliability of results and probably underlie their heterogeneity. We highlight perspectives and recommendations for future works on MR in psychiatry.

## Introduction

In the 2017 Global Burden of Disease Study, MDD was the third leading cause of nonfatal health problems, affecting more than 300 million people worldwide [1]. The World Health Organization (WHO) lists MDD as the largest single cause of disability worldwide and the leading cause of death by suicide, with approximately 0.8 million deaths annually [2]. MDD is a complex disease that is primarily influenced by external environmental variables, hereditary factors (approximately 40%) and particularly stressful experiences. Numerous epidemiological studies assessing the etiology of MDD have established a solid evidence base for disease prevention. For instance, one review revealed that sociodemographic factors, psychological issues, coexisting chronic conditions, and lifestyle factors are linked to the likelihood of MDD [3]. To prevent MDD, it is essential to identify modifiable causal risk factors. Randomized controlled trials (RCTs) continue to be the gold standard for determining causation, but they are costly, time-consuming, and prone to failure [4]. The causal inference of a single biomarker may also be affected by RCTs that frequently include multi-effect interventions (such as medications that change multiple biomarkers). Finally, RCTs may not be generalizable to the entire population; exposures (such as smoking) are unethical, and RCTs are prevented by extended lag times between exposure and disease [5]. Confounding variables and reverse causality hinder our understanding of the complex risk factors underlying diseases, although the results of observational research offer preliminary evidence of potential exposures linked to diseases [6]. Therefore, it remains unclear whether the factors observed in previous observational studies are causally related to the risk of MDD.

**Fig. 1:**
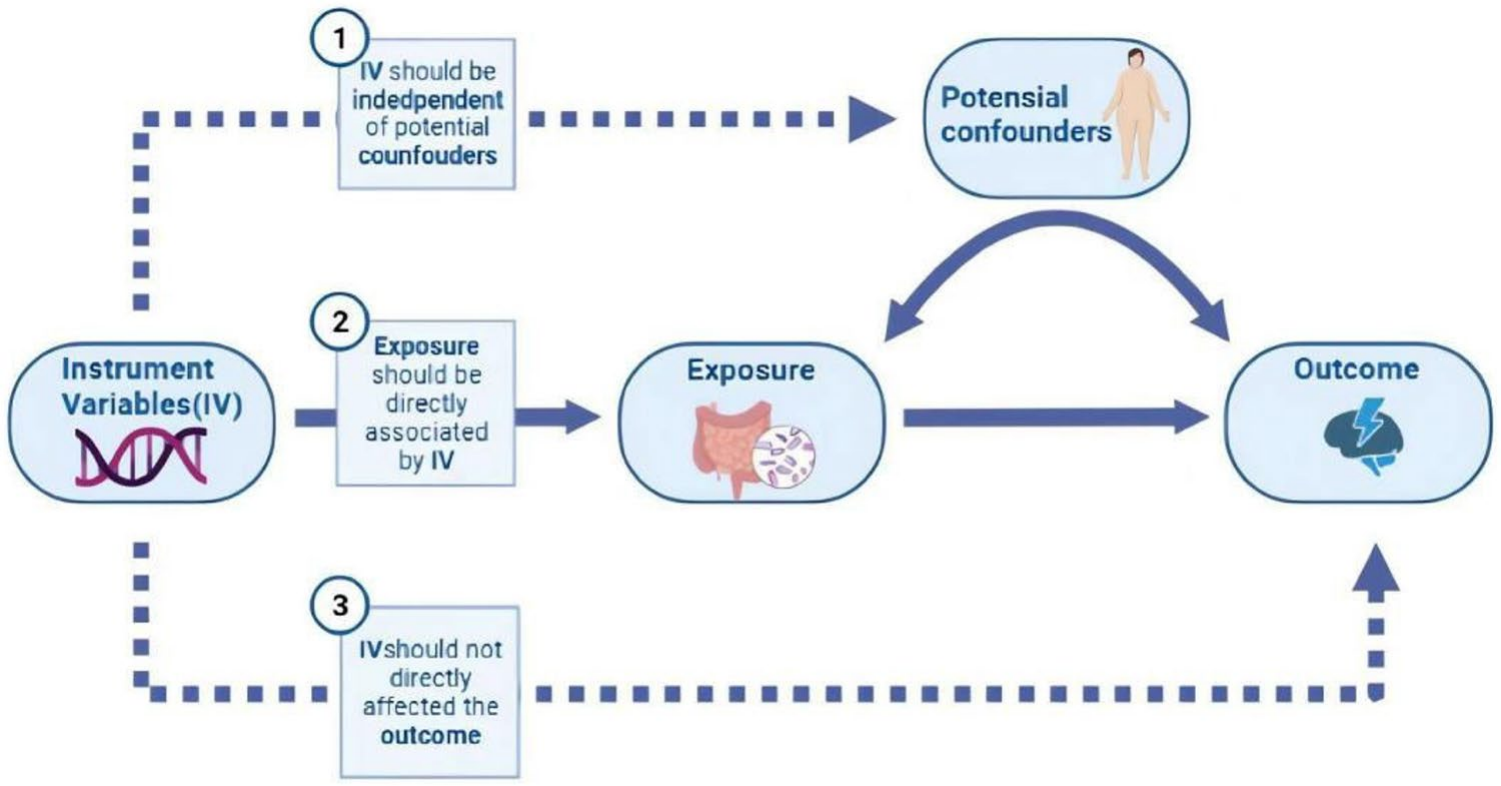
The basic principles and core assumptions of Mendelian randomization (MR). Correlation hypothesis: IV must be strongly correlated with exposure. Independence assumption: IV must be independent of confounding factors. Exclusivity assumption: IV will only affect the outcome through exposure

**Fig. 2:**
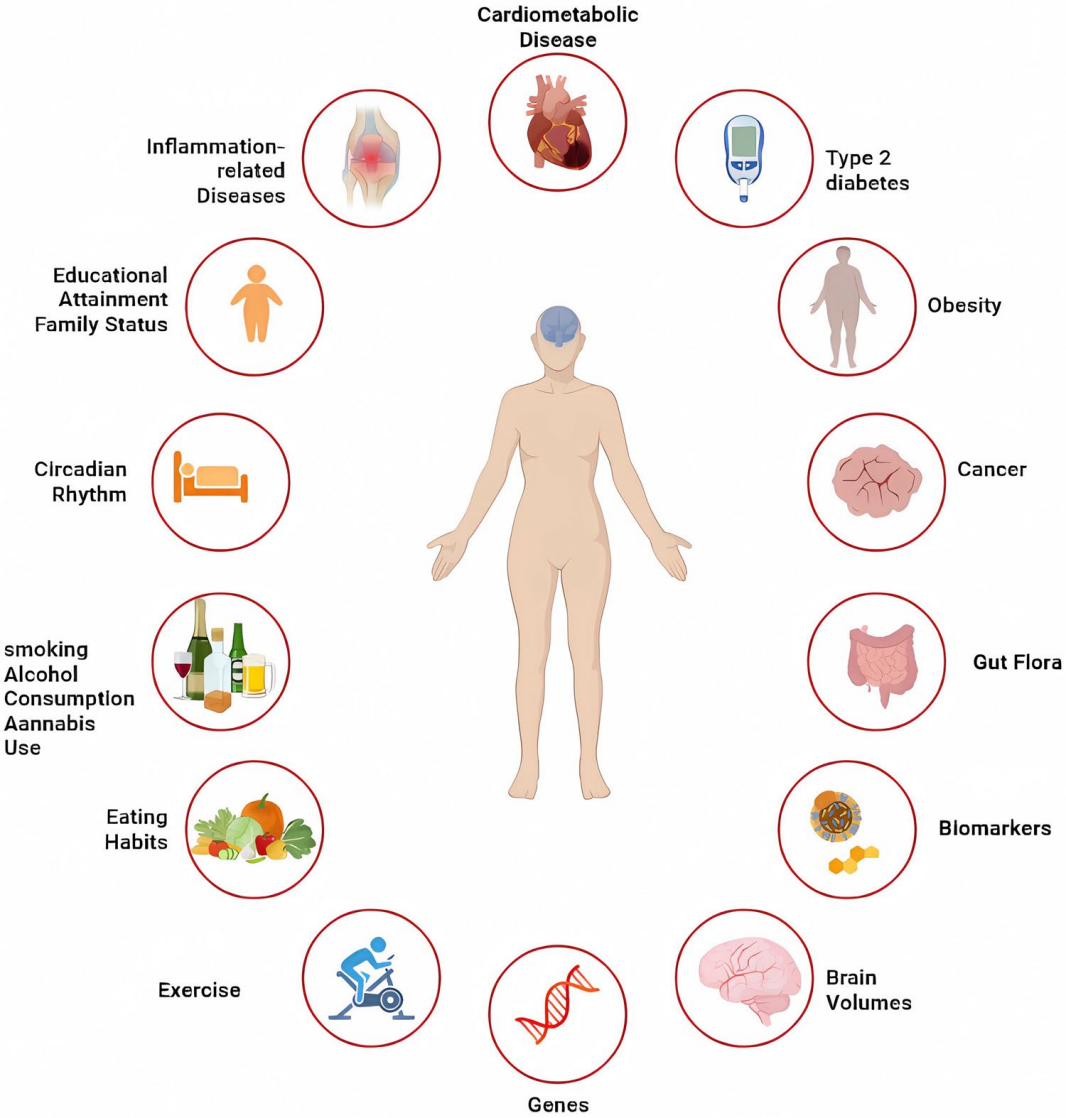
Cigarette smoking, excessive alcohol consumption, marijuana use, elevated circulating TG, C-reactive protein(CRP) and IL-6 levels, decreased DHA and increased omega-6: omega-3 fatty acid ratios, BMI, AAM, shorter telomere lengths, intestinal flora, type 2 diabetes mellitus, and breast cancer were associated with increased risk of major depressive disorder (MDD). In contrast, higher carbohydrate and beef intake, physical activity, higher education, early circadian preference, and living with a spouse or partner were associated with a lower risk of MDD. MR analyses have not yet established a causal relationship between genetic prediction of the prevalence of CAD, MS, types of cancer other than breast cancer, AD, JIAU, or periodontitis

MR is an epidemiological method that strengthens causal inferences using genetic variation as an instrumental variable [7]. The rationale for the MR and the required instrumental variable (IV) assumptions are as follows:(1) The IV is closely related to exposure factors, (2) the IV is independent of any of the unmeasured confounders, and (3) the effect of IV on the results is mediated only through exposure (Fig. 1). During meiosis, alleles are randomly assigned according to Mendel's second law and are thus usually independent of environmental and self-adoption factors; thus, MR is considered to be less influenced by confounding factors. Therefore, in this review, we aimed to summarize the evidence for MDD potential causal risk factors by integrating published MDD-related MR studies (Table1) and reflecting on the strengths and limitations of future prospects for MDD-related MR studies (Fig. 2).

**Table 1:** Notable Mendelian randomization studies in MDD

Exposure	Outcome	Primary ancestry	No. of SNPs	Source	N	Estimate(95% CI)	P	Refs
Smoking initiation	MDD	European	20	UK Biobank	249, 171	0. 324(CI 0. 128–0. 519)	0. 001	Barkhuizen et al., [12]2021
Smoking	MDD	European	378	GSCAN	599, 289	1. 99(CI 1. 71–2. 32)	0. 001	Wootton et al., [13]
Smoking initiation	MDD	European	353	GSCAN	1, 232, 091	1. 38(CI 1. 31–1. 45)	2. 3 × 10^−38^	Yuan et al., [14]2020
Lifetime Smoking	MDD	European	126	UK Biobank	462,690	2. 01(CI 1. 71–2. 37)	0. 001	Galan et al., [15]
Smoking	MDD	Norwegian	56,664	HUNT	53, 601	1.02 (CI 0.98–1.06)	0. 312	Bjørngaard et al., [16]
Smoking	MDD	European	1882	ALSPAC	6294	0.94 (CI 0.84–1.05)	0.25	Lewis et al., [17]
Alcohol consumption	MDD	East Asian	Unclear	CCGS	476	− 0.127 (CI: − 0.253, − 0.001)	0. 048	Zhu et al., [20]
Alcohol consumption	MDD	East Asian	Unclear	BeTwiSt	1,608	F = 1.39	0. 24	Chao et al., [21]
Carbohydrate intake	MDD	European	7	SSGAC	268, 922	0. 42 (CI 0. 28 -0. 62)	1. 49 × 10^−5^	Yao et al., [24]
Beef intake	MDD	European	9	PGC, UKBB	449, 210	−1. 252	0. 036	Chen et al., [27]
Cannabis use	MDD	European	19	UK Biobank	126, 291	1. 016 (CI 0. 994–1. 037)	0. 147	Hodgson et al., [29]
Accelerometer-based activity	MDD	European	9	UK Biobank, PGC	611, 583	0. 74 (CI 0. 59–0. 92)	0. 006	Choi et al., [31]
Morningness	MDD	European	268	UK Biobank	451, 025	0. 92 (CI 0. 88–0. 97)	Unclear	O'loughlinet al, [35]
Education level	MDD	European	663, 178	UK Biobank	170,756	0. 78 CI (0. 74–0. 83)	5. 21 × 10^–19^	Yuan et al., [38]
Education attainment	MDD	European	365	UK Biobank	142, 646	0. 72 (CI 0. 64–0. 80)	2. 06 × 10^–9^	Cai et al., [39]
Living with a partner Parents of 1 child Parents of 3 children	MDD	European	51	UK Biobank	52, 078	0. 67 (CI 0. 62–0. 74) 1. 17 (CI 1. 07–1. 27) 1. 11 (CI 1. 03–1. 20)	Unclear	Giannelis et al., [43]
S-25OHD	MDD	European	6	PGC	59, 851	1. 02 (CI 0. 97–1. 08)	0. 44	Michaëlsson et al., [47]
S-25OHD	TRD AD	European	110	UK Biobank	1,981 2,101	1. 12(CI: 0. 78–1. 31) 1. 04 (CI 0. 80–1. 36)	0. 567,396	Arathimos et al., [48]
S-25OHD	DS BD	European	6	SUNLIGHT	79,366	B = 0. 025, SE = 0. 038 B = 0. 020, SE = 0. 012	0. 52 0. 10	Libuda et al., [49]
S-25OHD	MDD	European	6	SUNLIGHT,UK Biobank,PGC	79,366	0. 97(CI 0. 90–1. 05)	0. 67	Mulugeta et al., [50]
TG	DS DSH	European	72 223	UK Biobank	188,577 480, 359	0. 0346(CI 0. 0114–0. 0578) 2. 514(CI 1. 579–4. 003)	3. 48 × 10^−3^ 1. 02 × 10^−4^	So et al., [51]
Omega-6:omega-3	MDD	European	18	UK Biobank	29,757	B = 0. 062, SE = 0. 026	0. 031	Davyson et al., [53]
1-Linoleoyl glycerophosphoethanolamine	MDD	European	Unclear	KORA F4 Twins	75 607	1.23(CI 1.09–1.38)	0.01	Yang et al., [54]
EPA DHA	MDD	European	106	ALSPAC	3,397	1. 07(CI 0. 99–1. 15) 1. 08(CI 0. 98–1. 19)	0. 083 0. 102	Sallis et al., [55]
CRP	MDD	European	Unclear	UK Biobank	147, 478	06(CI 1. 05–1. 08)	0.001	Milaneschi et al., [60]
IL-6	MDD	European	Unclear	PGC	135,458	1. 08(CI 1. 03–1. 12)	0.017	Perry et al., [61]
CRP IL-6	MDD	European	128	UK Biobank	204,402 500,199	(genetic correlation range, 0. 152–0. 362),SE = 0. 035	0. 001 0. 01	Kappelmann et al., [62]
IL-6	MDD	European	Unclear	UK Biobank	89, 119	1. 023(CI 1. 006–1. 039)	0. 006	Kelly et al., [63]
CRP	MDD	European	Unclear	DPR	78, 809	1. 28(CI 1. 23–1. 33)	0.001	Palmos et al., [64]
CRP	MDD	Danish	4	CGPS	78, 809	0.79(CI 0.51–1. 22)	0.03	Wium et al., [65]
Brain proteins	MDD	European	1,468	dPFC	307,353	Unclear	Unclear	Wingo et al., [66]
AAM	MDD	European	360	UK Biobank	807, 553	0. 96(CI 0. 94–0. 98)	0. 0003	Hirtz et al., [68]
Hippocampal volume	MDD	European	731, 536	UK Biobank	24,048	0. 98(CI 0. 95–1. 01)	0. 0850	Wigmore et al., [71]
DCC	MDD	European	3,725,946	PGC,UK Biobank	170,756	Unclear	Unclear	Li et al., [72]
TL	MDD	European	155	UK Biobank	1,628	− 0. 007(CI −0. 044–0. 029)	0. 700	Michalek et al., [74]
Bacilli class	MDD	European	Unclear	PGC,UK Biobank	2,076	1. 07(CI 1. 02–1. 12)	0. 010	Zhuang et al., [76]
BMI	MDD	European	35	PGC,UK Biobank	480, 000	0. 17(SD 0. 03)	1.0 × 10^−7^	Speed et al., [80]
BMI	MDD	European	73	PGC	48 791	1.18(CI 1. 09–1. 28)	0. 00007	Tyrrell et al., [81]
BMI	MDD	European	31	UK Biobank	2450	1.96(CI 0.03–3.90)	0.047	Jokela et al., [83]
BMI	MDD	European	321	MoBa	40, 949	0.26(CI −0.01–0.52)	0.060	Hughes et al., [84]
BMI	MDD	European	6	UK Biobank	339, 224	0.017 ~ 0.050	0.040	He et al., [85]
BMI	MDD	European	Unclear	CoLaus|PsyCoLus	3,350	SE = 0.23	0.004	Pistis et al., [86]
BMI	MDD	European	32	GENDEP	811	70.3(CI 70.18–0.13)	0.73	Hung et al., [87]
T2D	MDD	Chinese	34	Communities	11,506	1.21( CI 1.07–1.37)	0.0003	Xuan et al., [90]
T2D	MDD	Scotland	11	SFHS	19,858	rG = 0.0278	0.79	Clarke et al., [91]
MS	MDD	European	102	PGC, UK Biobank	246, 363	1.00(CI 0.99–1.01)	0.51	Harroud et al., [95]
AD	MDD	European	Unclear	UK Biobank	10,788	1.004	0.5681	Baurecht et al., [102]
JIAU	MDD	European	8	UK Biobank	807,553	1.001(CI 0.997–1.006)	0.581	Zhang et al., [104]

*MDD* major depressive disorder, *TRD* treatment-resistant depression, *AD* atypical depression, *DS* depressive symptoms, *BD* broad depression, *DSH* deliberate self-harm, *SNP* single nucleotide polymorphism, 95% CI 95% confidence interval

## Methods

### Search strategy and selection criteria

**Fig. 3:**
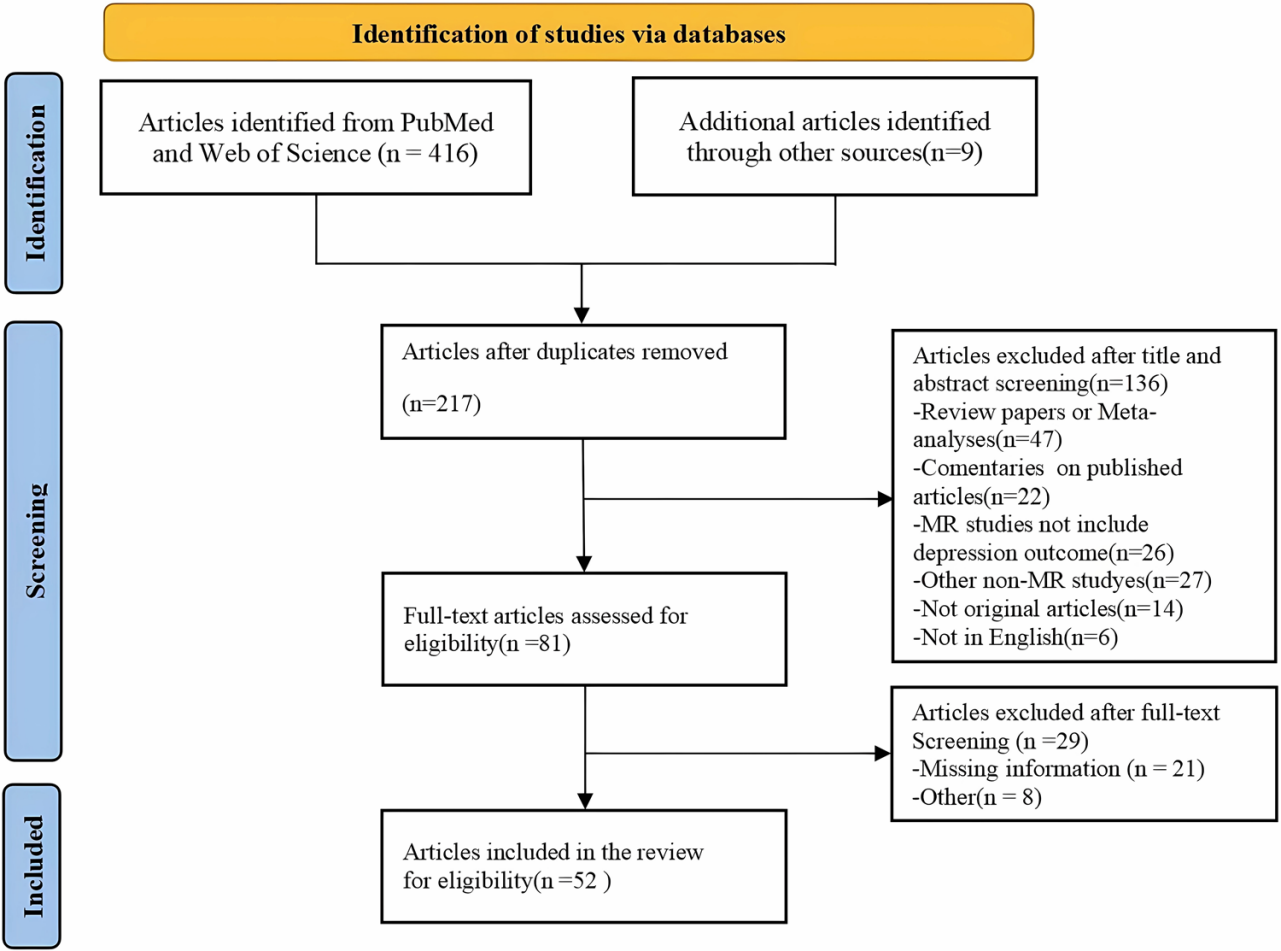
Flow diagram of studies included in systematic review

In accordance with the Preferred Reporting Items for Systematic Reviews and Meta-Analyses (PRISMA) criteria, this review was conducted by searching the PubMed and Web of Science databases for all articles published between the beginning of the databases and August 4, 2023. Search terms such as "Mendelian randomization" or "depression" or "genetic instrumental variable" or terms related to MR and depression were used in conjunction with Mesh word lists and related free text terms. Based on the reference lists of the included studies and pertinent reviews, we also found papers that might be suitable, and the selection process is documented in the PRISMA flow diagram (Fig. 3).

The reference library received all literature found through a systematic search, where duplicates were found and eliminated. Three authors separately assessed the literature using the COVIDENCE system evaluation program; JM and FY individually analyzed each article's title and abstract before confirming its eligibility for inclusion by reading the complete text. In case of any disagreements, a third researcher (YH) was consulted to determine whether to include or exclude papers.

Studies were accepted if they satisfied all three of the following requirements: (1) use of an MR design, (2) exposure to a risk factor for anything (potential depression or another disease risk factor), and (3) risk for something (both depression and non-depression). Additionally, we disregarded papers that were any of the following: (1) not human research; (2) written in a language other than English; (3) conference abstracts, reviews, or editorials; or (4) without complete text available.

### Data extraction

One author (YH) extracted the data and typed them into appropriate forms, while another author (XF) independently examined them. Each MR study's exposure, results, single nucleotide polymorphism count, data source, sample size, odds ratio (OR) and/or risk ratio (RR) (95% CI), and p-value for the MR analysis were collected.

### Quality assessment

Two reviewers (YH and XF) separately evaluated the caliber of the chosen research; any disparities were discussed, and a third reviewer (JM) settled any differing opinions. The risk of bias in MR studies cannot be assessed using a standardized tool. Therefore, to evaluate the quality of the included studies, we examined whether the three MR hypotheses had been validated, and if so, the procedures used.

## Result

### Lifestyle and MDD

#### Smoking

Epidemiological studies revealed that smoking often coexists with MDD [8]. The mechanism of smoking–depression co-morbidity remains unclear. One hypothesis is that both smoking and MDD are caused by common underlying genetic and environmental factors [9]. Another hypothesis is that there is a causal relationship between smoking and MDD; for example, depression increases the risk of smoking, and smoking increases the risk of developing MDD. Some studies suggest that individuals with increased depression exhibit increased smoking behavior as well [10]. In contrast, an MR study by Trine et al. revealed that daily smoking was associated with an increased risk of MDD [11]. Even after controlling for the genetic effects of covariates (marijuana and alcohol use, insomnia, and risk-taking behavior), Wikus et al. found significant conditional genetic associations between smoking frequency, current smoking status, and MDD. The MR results also supported a causal effect of smoking initiation on MDD [12]. The results of a two-way MR analysis by Wootton et al. revealed that smoking was a risk factor for MDD, lifetime smoking and initiation were consistent, and genetic susceptibility to MDD increased smoking behavior [13]. The results of the MR study by Yuan et al. revealed that initiation of smoking was positively associated with six psychiatric disorders including MDD [suicide attempts, post-traumatic stress disorder(PTSD), schizophrenia, bipolar disorder, MDD, and insomnia)]. In addition, association between the liability to depression and increased risk of smoking initiation also significant in a positive direction [14]. An MR study by Galan et al. suggested that the genetically predicted lifetime smoking index is associated with the risk of depression, and that the IL-6 pathway may be partially involved in the comorbid process of smoking and depression [15].^.^ A consistent association between any SNP and anxiety or depression in smokers has not been observed; however, the rs1051730 SNP variant in the nicotinic acetylcholine receptor gene cluster on chromosome 15 was used as an instrumental variable for the smoking phenotype [16]. Similar findings were found in an MR study by Sarah et al., who discovered that among women who smoked before becoming pregnant, the more the women who carried the 1,051,730 T allele smoked, the less inclined they were to give up smoking during pregnancy, and the less inclined they were to be depressed at 18 weeks of pregnancy. However, there was no evidence to support the claim that smoking causes depression [17]. These contradictory results imply that MR analysis of the causative relationship between smoking and MDD risk needs further improvement, and that further high-quality cohort studies are required to clarify this association.

#### Alcohol consumption

Excessive alcohol consumption is a common problem in patients with MDD. Observational studies have demonstrated that moderate alcohol consumption improves mental health and reduces stress [18]. However, a meta-analysis revealed that excessive alcohol consumption in MDD was associated with a more severe course of MDD, increased risk of relapse, reduced likelihood of MDD recovery, increased risk of suicide/death, and deterioration in social functioning [19]. According to an MR study, alcohol consumption was strongly associated with a lower risk of MDD, and the association persisted even after controlling for factors such as income, smoking, and parental drinking habits, as well as omitting heavy drinkers and previous drinkers [20]. In contrast, an MR study by Chao et al. used the acetaldehyde dehydrogenase 2 (ALDH 2) rs671 polymorphism as an instrumental variable for the drinking phenotype and found no significant correlation between alcohol consumption and anxiety or depression [21]. Therefore, future research should concentrate on specific SNPs in MDD to ascertain whether and how a particular SNP contributes to MDD.

#### Eating habits

The link between carbohydrate consumption and MDD has recently attracted considerable interest. According to a number of observational studies, carbohydrates have been linked to a lower risk of MDD because they boost the brain's uptake of tryptophan which stimulates serotonin synthesis and elevates mood [22]. Some trials have found little effect and even an increased risk of depression when the relative carbohydrate intake is increased [23]. There is still debate regarding the connection between carbohydrates and MDD. According to an MR investigation that demonstrated a causal association between higher relative carbohydrate intake and a reduced risk of MDD, the protective effect of relative carbohydrate intake on MDD persisted across different dietary composition circumstances after several corrections [24].

Despite research indicating the potential advantages of healthy eating habits for the management and prevention of MDD symptoms, data on the impact of specific foods on MDD are limited. For instance, a meta-analysis of observational studies found no correlation between the frequency of MDD and increased meat consumption [25]. Most studies have shown that vegetarians and vegans are more likely to develop MDD [26]. According to the MR analysis by Chen et al., eating beef and grains may reduce the risk of developing MDD, whereas eating non-oily fish may increase the risk [27]. An RCT is required to confirm the relationship between carbohydrate intake and MDD. *Cannabis use.*

Numerous theories have been proposed regarding the mechanisms linking cannabis use and MDD: that the two are causal, or that they may be caused by common factors (genetic or environmental). For instance, cannabis use and MDD have a statistically significant genetic correlation [28]. Similarly, a 2019 MR study could not establish causality between cannabis use and MDD, but found strong clinical and genetic connections between the two [29].

#### Exercise

Exercise interventions are a promising approach for treating depressive symptoms, and these interventions do not have side effects or high costs of antidepressant medications and psychotherapy. According to a meta-analysis, those who engage in high levels of physical exercise had a 17% lower prevalence of MDD than those who engage in low levels of physical activity [30]. According to the findings of this MR study, there is a protective link between objectively measured (as opposed to self-reported) physical activity and MDD. These findings lend credence to the idea that increased physical activity may be a successful preventive strategy [31]. Although the outcomes of this MR study are somewhat in agreement with those of observational research, more convincing evidence is required to support them.

#### Circadian rhythm

Circadian rhythm is an approximately 24-h periodic physiological process found in most organisms, including humans [32]. A cross-sectional study reported that individuals with early circadian preferences had fewer MDD symptoms [33]. A recent MR investigation found 351 genetic variations linked to circadian preferences, and the findings revealed that early circadian preference is causally linked to improved subjective well-being, reduced prevalence of schizophrenia, and current MDD symptoms [34]. A similar protective effect of an early circadian preference on MDD and enhanced health was observed in an MR study by O'Loughlin et al. [35]. However, this study did not examine whether the findings were sex-specific or whether it employed more thorough mental health measures.

#### Educational attainment

Educational attainment is a determinant of a healthy society and is a modifiable risk factor for MDD [36]. Observational studies have revealed a protective effect of high educational attainment on severe MDD [37]. According to an MR study by Yuan et al. [38], a genetic propensity for higher educational attainment was linked to a lower incidence of severe MDD [38]. A similar causal link between higher educational attainment and decreased MDD incidence was found in an MR study by Cai et al. [39].

#### Family status

The prevalence and severity of MDD have been linked to family status, including marital status, cohabitation with a spouse or partner, and parental status [40]. For instance, a comprehensive meta-analysis of research on older Chinese adults revealed that unmarried (including divorced, unmarried, and widowed) individuals have a significantly higher prevalence of MDD [41]. One study found that depressive symptoms decreased with an increase in the number of children [42]. Living with a spouse or partner was found to be substantially related to lower lifetime odds of depression, whereas parents of one child and three or more children had greater lifetime odds of depression than people without children [43].

### Biomarkers and MDD

#### 25(OH)D

Vitamin D receptors are widely distributed throughout the human brain, including in the limbic structures, and may be involved in mood regulation. The active form of vitamin D, osteotriol, can affect the synthesis of catecholamines and 5-hydroxytryptamine associated with mood disorders, thereby altering emotional behavior [44]. A meta-analysis revealed a correlation between vitamin D levels and depression in elderly [45]. Another cross-sectional study confirmed a negative correlation between 25(OH)D concentration and MDD [46]. In contrast, an MR study revealed that genetically predicted 25(OH)D concentrations are not associated with MDD [47]. There is some evidence of a weak correlation between 25(OH)D and MDD (TRD) and atypical MDD (AD) in the MR analysis by Arathimos et al. Genetic evidence does not support a causal relationship between vitamin D and TRD or AD [48]. An MR study found no significant correlation between 25(OH)D concentration and depressive symptoms (DS) or generalized depression (BD) [49]. In MR inverse variance weighting (IVW) from the UK Biobank, there was no correlation between genetically determined serum 25(OH)D levels and MDD, in contrast to genetic susceptibility to MDD, which is associated with lower 25(OH)D concentrations [50]. Therefore, the causal relationship between 25(OH)D levels and MDD requires further investigation.

#### Blood lipids

Previous research has revealed that decreased membrane cholesterol may limit the number of serotonin receptors, increasing the risk of suicide, and that lower peripheral cholesterol levels may result in a decrease in brain 5-hydroxytryptamine. Triglycerides (TG) and depressive symptoms (DS) have been found to be causally related using MR analysis [51]. However, there was no relationship between the lipoprotein fraction or ApoE genotype and depression scores in blood total cholesterol of older men [52]. Therefore, to establish a clear causal link between lipids and MDD, high-quality RCT trials and improved MR analyses are required.

#### Fatty acids

Docosahexaenoic acid (DHA), an omega-3 fatty acid, has long been known to affect behavior and brain function; however, its precise association with MDD is uncertain. According to the findings of an MR investigation, MDD may be caused by a decrease in DHA levels and an increase in the Omega-6: Omega-3 fatty acid ratio [53]. The causative relationship between 1-oleoylglycerol phosphate ethanolamine and MDD was demonstrated in the MR study by Yang et al. [54]. Although there is some tenuous support for a possible link between omega-3 fatty acids and prenatal MDD, a causality could not be established [55].

#### CRP and IL-6

There is growing evidence of the involvement of the immune system in the pathogenesis of MDD. Elevated C-reactive protein (CRP) levels are present in approximately one-quarter of patients with MDD and longitudinally predict the onset of MDD-like symptoms [56]. Previous studies have generally combined indicators of MDD, although the condition itself is phenotypically variable. According to recent studies, inflammatory alterations have been linked to more "atypical" energy-related symptoms of MDD, including increased sleep, increased hunger and weight gain, lethargy, and lead poisoning paralysis [57–59]. According to a study by Milaneschi et al., elevated CRP levels are linked to MDD symptoms, such as altered appetite, trouble sleeping, and exhaustion. Higher IL-6 levels in NESDA have also been linked to decreased appetite, loss of pleasure, exhaustion, and sleep issues, suggesting that the IL-6/IL-6 R signaling pathway may be causally related to MDD [60]. Similarly, MR evidence from Perry et al. supports a causal relationship between IL-6 and MDD [60]. In addition, high CRP levels are associated with loss of pleasure, fatigue, appetite changes, and discomfort, and the upregulation of genes involved in IL-6 signaling is associated with suicidal ideation [61]. The results of the MR analysis by Kelly et al. provide evidence for IL-6 signaling in MDD, and this relationship may be consistent with a potential mechanism of reduced classical signaling or increased trans-signaling [60, 62]. However, the MR analysis performed by Palmos et al. supported the lack of a causal link between CRP and MDD [63]. Similarly, a 2014 MR study of the general Danish population revealed that, in contrast to genetically elevated CRP levels, elevated plasma CRP levels are associated with an increased risk of depression and psychological distress. This shows that there is no direct causal relationship between CRP and MDD or psychological distress and that underlying inflammation caused by other diseases may be a more rational explanation for this association [64]. Therefore, experimental investigations are required to examine the mechanisms and efficacy of immunotherapy for MDD, and to further assess the causal link between inflammatory variables and MDD.

#### Brain proteins

Research on MDD has explored the genetic, epigenetic, and transcriptional factors; however, few studies have directly explored brain proteins. To identify brain proteins that are causally associated with MDD, Wingo et al. performed an MR analysis of proteome-wide association studies of MDD, resulting in the identification of 25 brain proteins that were causally associated with MDD, 20 of which have not been previously reported to be associated with MDD [65]. These findings provide new brain protein targets for the further exploration of the mechanisms and treatment of MDD.

### Other risk factors and MDD

#### Age at menarche

Sex differences in MDD prevalence occur only in adolescence, with girls having twice the prevalence as boys [66]. To better understand girls' adolescent-related mental health, the MR study by Raphael et al. conducted a generalized MDD risk analysis using 360 SNPs associated with age at menarche (AAM) as an instrumental variable. The results revealed a significant causal relationship between AAM and the risk of depression [67].

#### Brain volumes

In studies of patients with MDD conducted in cross-sections, low brain volumes are typically found. A meta-analysis of subcortical brain volumes revealed a significant correlation between MDD and reduced hippocampal volume [68]. A reduction in the hippocampal volume has also been linked to MDD [69]. In contrast to the other six brain regions, hippocampal volume was positively correlated with MDD according to an MR study by Wigmore et al. However, there was no causal relationship between hippocampal volume and MDD. Additionally, it has been demonstrated that hippocampal volume and MDD may have hereditary similarities [70].

#### Genes

To identify the netrin 1 receptor (DCC) gene as a risk factor for depression, Li et al. combined information from GWAS, DLPFC expression quantitative trait loci (eQTL), and enhancer–promoter physical linkage investigations. This study indicates a significant link between depressive episodes and DCC, and hypothesizes that DCC may play a role in the molecular processes of synaptic plasticity, axonal guidance, circadian rhythms, and long-duration learning associated with MDD. This further supports the notion that DCC is a key depression-susceptibility gene, and may be a promising target for brand-new antidepressants [71].

#### Telomere length

Shorter telomeres indicate older cells or older people. Telomere length (TL) is a marker of "cell age" or "biological age." According to earlier research, patients with MDD and shorter leukocyte TLs age biologically faster [72]. However, this reverse causality has not yet been examined. In their MR investigation, Michalek et al. investigated the possibility that a genetic predisposition to shorter telomere length (TL) could lead to the onset of MDD earlier than expected. Researchers have shown, but to a lesser extent, that the consequences of late biological aging enhance the probability of childhood-onset recurrent MDD using the rs10936599 SNP [73]. Thus, telomerase may represent a promising therapeutic target for the treatment of childhood MDD. Larger cohort studies are warranted in the future.

#### Gut flora

According to mounting evidence, short-chain fatty acids (SCFA), neurotransmitters, and other bacterial metabolites may be crucial components of microbiota–host interactions that control brain activity and behavior [74]. Therefore, it may be clinically useful to understand the mechanics of the gut–brain axis in MDD. The results of an MR investigation indicate that Bacillus species are linked to a high risk of MDD [75], which can be explained by the possibility that Bacillus species are involved in the metabolism of dopamine, leading to the emergence of MDD symptoms [76].

### Diseases and MDD

#### Obesity

A body mass index (BMI) of 30 kg/m^2^ or more in an adult is considered obese, which is a severe global public health issue [77]. Obesity is a recognized risk factor for heart disease, type II diabetes, several malignancies, and a shorter lifespan. In addition, research has connected obesity to MDD. The results of an MR study by Ding et al. confirmed that all three obesity levels were positively associated with MDD and shared a common genetic locus [78]. A 2019 MR investigation identified height (short stature) and adiposity as causal risk factors for MDD, but could not conclusively demonstrate a link between non-adiposity and MDD risk [79]. A 2016 MR study found that a higher genetically predicted BMI was associated with an increased risk of MDD, particularly in women [80]. Genetic evidence from an MR study by Mulugeta et al. also suggested an association between MDD and high BMI [81]. Similarly, a 2012 MR study showed that higher adolescent and adult BMI predicted higher adult depressive symptoms [82]. A within-family MR study suggested that children’s BMI increased their depressive symptoms, maternal BMI may independently affect children's depressive symptoms, and there is limited evidence of the effect of paternal BMI on children's MDD symptoms [83]. An MR analysis by He et al. found a causal relationship between BMI and depressed mood and dependence on glutamine levels [84]. Another study found no evidence of a causal relationship between BMI and the likelihood of atypical symptoms or MDD [85]. A causal link between higher BMI and severe MDD was not supported by Chi et al. 's MR study [86]. These inconsistent results may be attributed to the heterogeneity of MDD.

#### Type 2 diabetes mellitus

Type 2 diabetes mellitus (T2 D) is significantly comorbid with MDD, and MDD is twice as likely to occur in individuals with T2 D as in those without T2 D [87]. A meta-analysis showed that patients with T2 D have a 15%–24% higher risk of developing depressive symptoms than those without T2 D [88]. Shared environmental and genetic risk factors have been hypothesized to underlie the co-morbidity of MDD and T2 D. The results of the MR analysis by Xuan et al. suggested a causal relationship between genetically determined T2 D and MDD [89]. However, no evidence of genetic overlap between MDD and T2 D was found in the MR analysis by Clarke et al. Environmental factors may better explain the co-morbidity of T2 D and MDD [90].

#### Cardiovascular disease

The association between cardiovascular disease (CVD) and MDD is multifaceted and likely bidirectional. On the one hand, a higher risk of cardiac events may be linked to the presence of depressed symptoms [91]. On the other hand, acute myocardial infarction (AMI) patients have a nearly three times greater prevalence of psychotic depression than the general population [92]. The MR analysis provides evidence that genetically increasing odds of MDD are causally associated with increased risk of CAD and MI, but reverse causation of CVD on MDD is not detected [93].

#### Multiple sclerosis

MDD is common in patients with multiple sclerosis (MS) and can accelerate disability progression. The increased mortality in patients with MS and MDD co-morbidity compared to those with MDD or MS alone suggests that MDD and MS share a common genome or environmental factors, or both. However, a bidirectional MR analysis by Harroud et al. did not support a causal relationship between genetic predisposition to MDD and MS susceptibility, and vice versa [94]. Currently, studies of MDD and MS are limited to European populations. Therefore, the relationship between MDD and MS requires further investigation.

#### Cancer

Another typical co-morbidity among patients with cancer is MDD. According to previous reports, patients with cancer have an increased risk of developing MDD [95]. Various types of cancers have varying rates of MDD. Breast, lung, oropharyngeal, and pancreatic malignancies are also frequently associated with MDD [96]. Patients with cancer are more likely to experience depression for several reasons such as inadequate pain management and chemotherapy drug use. The relationship between various cancer types, MDD, and its specific processes has not yet been clarified. There was no evidence of a causal relationship between MDD and any other type of cancer except breast cancer in a bidirectional MR analysis by Zhu et al. However, this finding does not indicate the absence of a causative relationship [97].

#### Inflammation-related diseases

Atopic dermatitis (AD), the most prevalent chronic inflammatory skin condition, has a complicated pathogenesis that includes genetic predispositions and environmental causes [98]. A case–control study using data from the UK Clinical Practice Study showed a significantly increased risk of depressive and anxiety events in patients with atopic dermatitis [99]. A meta-analysis confirmed the potential association between AD, MDD, and anxiety disorders [100]. Baurecht et al. found no evidence that atopic dermatitis is associated with the risk of MDD or anxiety [101].

Juvenile idiopathic arthritis (JIAU) is a pediatric rheumatic autoimmune arthritis that manifests before the age of 16 years and lasts for at least six weeks [102]. However, the processes underlying the association between JIAU and MDD remain unclear. Using a bidirectional MR analysis, Zhang et al. demonstrated that there are insufficient data to establish a link between JIAU and the bidirectional development of severe MDD [103].

Periodontitis originates from dysbiosis of the oral microbial community caused by key pathogens such as Porphyromonas gingivalis, which induces an inflammatory response through the release of lipopolysaccharides and downregulation of neutrophil recruitment [104]. These inflammatory signals propagate through humoral, cellular and neural pathways and reach the brain to trigger neuroinflammation, which can also induce MDD [105, 106]. A bidirectional MR study did not support a common heritability or causal relationship between MDD and periodontitis [107]. Therefore, other well-designed prospective studies are needed to minimize observational study bias and use larger GWAS data for MR analysis.

## Opportunities and challenges

MR uses the IVs technique to analyze observational data, and IV is a genetic variety that offers trustworthy proof of a causal link between exposure and results. To assist in targeted gene treatment for MDD, large-scale GWAS are now being utilized to discover genetic loci linked to MDD features and symptoms. This enables the identification of preventable and changeable environmental risk factors using MR. In MDD research, MR can be used to examine the directionality of causality and relationships between phenotypes. Before applying MR to draw conclusions regarding the causal relationships between exposure and results, three assumptions must be made: genetic variations can also be linked to many exposures in this study owing to multivariate MR analysis, which can be utilized to find connections between various categorical exposure factors and related effects. MR analysis has already been used in an increasing number of studies and offers a distinct benefit in MDD exploration. Two examples include eliminating exposure factors with significant causality and investigating the causative significance of exposure factors with weak correlations.

Two issues plague MR research of MDD: first, it is difficult to obtain generalized genetic information from comparable ethnic groups for two-sample MR. For several races and ethnicities, there are no large-scale GWAS of MDD or insufficient genetic information on the disorder. Additionally, the UK Biosample Repository database has been the primary source for most investigations and the impact of sample overlap should be considered when addressing these findings. Additionally, genetic information may not completely identify the exposure factors to establish a meaningful causal association. Although pleiotropy or horizontal pleiotropy has been assessed using MR-Egger regression intercepts, pleiotropy residuals, and global testing for outliers in IV, there may be substantial horizontal pleiotropy, which presents difficulties for hypothesis violation and biased statistical inference. However, there are certain issues associated with using MR approaches in practice, despite the fact that they can handle causally complex phenotypic interactions. When there is a bidirectional causal relationship between exposure and results, it is challenging to adopt bidirectional MR, because it is impossible to avoid selecting genetic variations associated with both exposure and outcome. Finally, to prevent covariation, multivariate MR has restrictions on the number of genetic variants; more genetic data are not allowed for some races and ethnicities, nor can it have more exposure than others.

## Conclusions

Therefore, MR analysis is important in the etiologic study of MDD. Overall, cigarette smoking, excessive alcohol consumption, marijuana use, elevated circulating TG, CRP, and IL-6 levels, decreased DHA and increased omega-6: omega-3 fatty acid ratios, BMI, AAM, shorter telomere lengths, intestinal flora, type 2 diabetes mellitus, and breast cancer were associated with increased risk of MDD. In contrast, higher carbohydrate and beef intake, physical activity, higher education, early circadian preference, and living with a spouse or partner were associated with a lower risk of MDD. MR analyses have not yet established a causal relationship between genetic prediction of the prevalence of CAD, MS, types of cancer other than breast cancer, AD, JIAU, or periodontitis. Despite some limitations, MR analyses can provide constructive insights for drug development and disease prevention, and provide valid guidance for observational studies and RCTs.

## Data Availability

Not applicable.
